# Tumor Neovascularization and Developments in Therapeutics

**DOI:** 10.3390/cancers11030316

**Published:** 2019-03-06

**Authors:** Yuki Katayama, Junji Uchino, Yusuke Chihara, Nobuyo Tamiya, Yoshiko Kaneko, Tadaaki Yamada, Koichi Takayama

**Affiliations:** Department of Pulmonary Medicine, Kyoto Prefectural University of Medicine, 465 Kajiicho, Kawaramachi-Hirokoji, Kamigyo-ku, Kyoto 602-8566, Japan; ktym2487@koto.kpu-m.ac.jp (Y.K.); c1981311@koto.kpu-m.ac.jp (Y.C.); koma@koto.kpu-m.ac.jp (N.T.); kaneko-y@koto.kpu-m.ac.jp (Y.K.); tayamada@koto.kpu-m.ac.jp (T.Y.); takayama@koto.kpu-m.ac.jp (K.T.)

**Keywords:** cancer therapy, neovascularization, angiogenesis, tumor microenvironment

## Abstract

Tumors undergo fast neovascularization to support the rapid proliferation of cancer cells. Vasculature in tumors, unlike that in wound healing, is immature and affects the tumor microenvironment, resulting in hypoxia, acidosis, glucose starvation, immune cell infiltration, and decreased activity, all of which promote cancer progression, metastasis, and drug resistance. This innate defect of tumor vasculature can however represent a useful therapeutic target. Angiogenesis inhibitors targeting tumor vascular endothelial cells important for angiogenesis have attracted attention as cancer therapy agents that utilize features of the tumor microenvironment. While angiogenesis inhibitors have the advantage of targeting neovascularization factors common to all cancer types, some limitations to their deployment have emerged. Further understanding of the mechanism of tumor angiogenesis may contribute to the development of new antiangiogenic therapeutic approaches to control tumor invasion and metastasis. This review discusses the mechanism of tumor angiogenesis as well as angiogenesis inhibition therapy with antiangiogenic agents.

## 1. Introduction

Vasculogenesis refers to the process by which vascular endothelial cells differentiate from endothelial precursor cells to form the lumen. Neovascularization refers to the process, whereby new blood vessels are formed from existing ones following endothelial cell proliferation and migration [1]. This process is essential during physiological angiogenesis, such as systemic blood supply in the fetal stage, luteinization related to postpartum menstrual cycle, and wound healing [2]. During tumor proliferation, oxygen and nutrients required for solid tumor growth are supplied from neighboring blood capillaries. However, because the diffusion distance of oxygen is 100–200 μm, for tumors to grow to ≥1–2 mm, generation of new blood vessels towards the tumor (i.e., neovascularization) is required [3,4]. Tumors located >100–200 μm from capillaries often encounter hypoxic conditions, which promote the expression of hypoxia-inducible factor-1 (HIF-1). HIF-1 induces the expression of angiogenic proteins, such as vascular endothelial growth factor (VEGF), epidermal growth factor, fibroblast growth factor (FGF), hepatocyte growth factor (HGF), and platelet-derived growth factor (PDGF), which then stimulate hypervascularization [5,6]. The sustained expression of these angiogenic factors results in abnormally structured angiogenic tumor vessels. Tortuous and dilated tumor vessels show increased vascular permeability and high interstitial pressure, further reducing blood perfusion and increasing hypoxic conditions in the tumor microenvironment [7,8,9]. Administration of angiogenesis inhibitors leads to tumor vascular normalization, a reduction in vascular permeability and interstitial fluid pressure, and an improvement in tumor perfusion. A normalized tumor vascular system with reduced hypoxic conditions not only augments the effects of radiotherapy and chemotherapy but also enhances antitumor immunity [10,11,12]. The findings can contribute to a new approach (i.e., the combination of angiogenesis inhibitors and immunotherapy) to further improve the overall survival of cancer patients. This review discusses the molecular mechanisms of tumor angiogenesis and outlines options for cancer therapy with antiangiogenic agents including combined immunotherapy.

## 2. Molecules Involved in Neovascularization

Neovascularization is regulated by a balance between angiogenesis-inducing factors and angiogenesis-inhibiting factors such as those outlined in Table 1. Here, we describe the molecules that induce angiogenesis and their mechanisms. Among angiogenesis-inducing factors, VEGF plays an important role in the initiation of angiogenesis. The VEGF family consists of five members, namely VEGFA, VEGFB, VEGFC, VEGFD, and placental growth factor (P1GF). VEGF signals are transmitted through three VEGF receptor tyrosine kinases: VEGFR1, VEGFR2, and VEGFR3 [8,13]. The VEGF family of proteins is the most critical factor for the induction of neovascularization. VEGF induces proliferation of endothelial cells, promotes cell migration, and decreases the rate of apoptosis. It also increases vascular permeability and promotes migration and circulation of other cells [13,14]. VEGFA and its receptor, VEGFR2, have major angiogenic effects [15]. Upon binding to the VEGF receptor on the vascular endothelial cell membrane, VEGF induces dimerization and autophosphorylation of the receptor and initiates a signaling cascade that activates a variety of downstream pathways. Phosphorylation of phospholipase C (PLC) γ activates the RAS/mitogen-activated protein kinase (MAPK) cascade via protein kinase C (PKC) activation and regulates gene expression and cell proliferation [16,17,18]. In addition, activation of the phosphoinositide-3-kinase (PI3K)/protein kinase B (AKT) pathway produces NO via AKT, suppresses apoptosis, and activates endothelial cell NO synthase, thereby enhancing vascular permeability [19,20,21,22]. VEGFR1 has a weak kinase activity and limits VEGFR2-induced angiogenic effects by regulating the amount of VEGFA that can be bound by VEGFR2 [23]. The following has been reported: (i) VEGFR3 and its ligand, VEGFC, are responsible for lymphangiogenesis; (ii) VEGFC and VEGFD contribute to tumor angiogenesis by binding to VEGFR2 and VEGFR3; (iii) VEGFR3 is expressed in the tip cells of tumor vessels [15,24].

Angiopoietins play a critical role in the maturation of blood vessels. Human angiopoietins consist of three ligands, Ang-1, Ang-2, and Ang-4. Ang-1 and Ang-2 are of critical importance in angiogenesis and are outlined hereafter. The Tie family of receptors includes receptor tyrosine kinases specifically expressed in the vascular endothelium. They include Tie1 and Tie2. Tie2 is activated by Ang-1, which is secreted by platelets and peri-endothelial cells; whereas Tie1 is an orphan receptor homolog of Tie2, whose expression enhances Tie2 activation [25]. The Ang-1/Tie-2 signaling pathway is specific for endothelial cells. Ang-1 binds to the Tie-2 tyrosine kinase receptor of endothelial cells, whose downstream phosphorylation activity stimulates cell survival by activating the PI3K-AKT pathway [26,27,28]. Furthermore, it contributes to the maturation of blood vessels by inhibiting the proinflammatory pathway initiated by nuclear factor-kappa B (NF-κβ) [26,27,28]. In contrast, in the absence of cell-cell adhesion, extracellular matrix-anchored Tie2 regulates angiogenesis via extracellular signal-regulated kinase (ERK) 1/2 signaling [29]. Ang-2 antagonizes Ang-1 activity and, in the presence of low levels of VEGF, leads to detachment of pericytes and regression of blood vessels. However, in the presence of high levels of VEGF, Ang-2 elicits an inflammatory response and destabilizes existing vessels. This, in turn, promotes angiogenesis and lymphangiogenesis by weakening the interaction between endothelial cells and pericytes and increasing endothelial cell migration [1,30,31].

## 3. Characteristics of Angiogenic Tumor Vessels

Angiogenesis-promoting factors such as VEGF induced by the tumor microenvironment (e.g., hypoxia), stimulate sustained and abnormal neovascularization [32,33]. The vessels formed during neovascularization are unlike those formed during wound healing and exhibit unusual morphological characteristics. In normal vessels, the distribution of arteries, capillaries, and veins is stable, and the vessels have an ordered hierarchical structure. In comparison, angiogenic tumor vessels are dilated and tortuous. Furthermore, vascular density and blood vessel diameter are not uniform [34,35]. A simple squamous epithelium, known as vascular endothelial cells, covers the lumen of capillaries, which is lined with pericytes and covered by the basement membrane. Angiogenesis promoting factors induce weakening and migration of vascular endothelial cell junctions and change the vascular wall structure [36,37]. Pericytes and vascular endothelial cell junctions between pericytes and vascular smooth muscle cells are also weakened, and the number of pericytes is reduced [38,39]. The basement membranes are multilayered and collagen IV thickness is uneven. Weakened cell junctions between endothelial cells and pericytes result in their infiltration into the tumor stroma [38,39]. The morphological abnormalities observed in tumor blood vessels raise the question of whether there are phenotypic differences at the molecular and functional levels between tumor endothelial cells (TECs) that line tumor blood vessels and normal endothelial cells. TECs express higher levels of proangiogenic genes such as VEGFR, VEGF, and EGFR. The Hu antigen, a neuronal protein identified in the serum of patients with small cell lung cancer and paraneoplastic encephalomyelitis/sensory neuronopathy, promotes TECs survival by stabilizing VEGF mRNA. TECs also up-regulates integrin αVβ3 and cause cytogenetic abnormalities [40]. Moreover, in comparison with normal endothelial cells, TECs have a high proliferative capacity, migratory ability, and angiogenic potential [41]. Additionally, cells showing stem cell/precursor cell-like properties have been reported in the TECs population, together with those originating from bone marrow-derived vascular endothelial progenitor cells and tissues derived from tissue stem cells [42]. Furthermore, a population expressing stem cell markers such as aldehyde dehydrogenase and having high angiogenic potential has also been reported [43]. ATP-binding cassette sub-family B member 1 (ABCB1) is the most well-known drug efflux transporter and TECs strongly expressing ABCB1 are resistant to drugs [44]. Importantly, cancer microenvironment factors such as hypoxia, are also thought to be involved in tumor vascular endothelial cell abnormalities, together with humoral factors derived from cancer cells and exosomes [45,46]. Tumor vascular endothelial markers are expressed in cancer cells when cultured under hypoxia or in a low-serum medium. Furthermore, Kubota et al. reported that an ataxia telangiectasia mutated kinase was strongly activated in immature vessels in response to the accumulation of reactive oxygen species, where it provided a defensive function [47]. More recently, Maishi et al. demonstrated that biglycan secreted by TECs induced intravascular invasion and metastasis of cancer cells and reported a new mechanism of cancer metastasis induction from tumor vessels [48]. As can be seen, various factors typical of the cancer microenvironment exert complex and diverse properties in tumor vascular endothelial cells.

## 4. Regulatory Mechanisms of Neovascularization

### 4.1. HIF-1α

**Figure 1:**
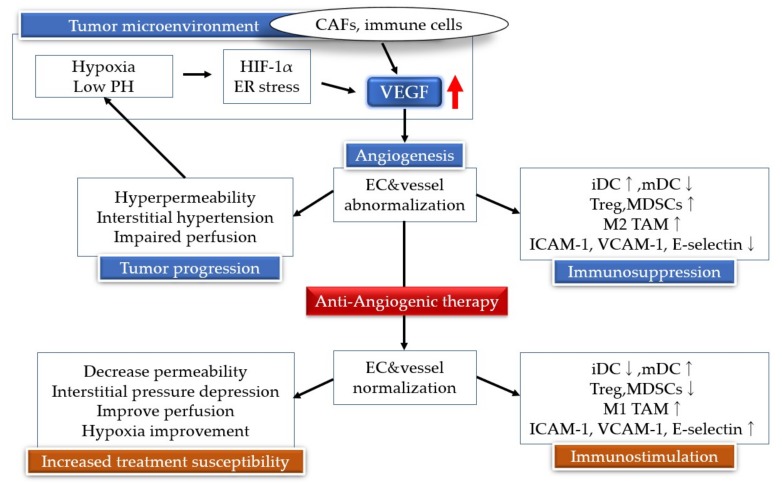
Hypoxia inducible factor (HIF) and vascular endothelial growth factor (VEGF) link the angiogenesis signaling pathways. Low oxygen tension (hypoxia) results in constitutive activation of the HIF pathway and VEGF. The tumor hypoxic environment leads to an immunosuppressive tumor microenvironment by inducing regulatory T cells (Tregs), myeloid-derived suppressor cells (MDSCs), and M2 tumor-associated macrophages (TAMs). Antiangiogenic therapy results in blood vessel regression by suppression of neovascularization, leading to tumor starvation and tumors falling into dormant states. CAFs, cancer-associated fibroblasts; iDCs, immature dendritic cells; mDCs, mature dendritic cells; ICAM-1, intercellular adhesion molecule 1; VCAM-1, vascular cell adhesion molecule 1.

Hypoxic conditions in the tumor microenvironment up-regulate angiogenesis inducing factors such as VEGF, PDGF, P1GF, and HGF. However, reactions activated by such a hypoxic environment are thought to be elicited primarily by HIF-1α [49,50]. HIF is a transcription factor heterodimer consisting of subunits HIF-1α and HIF-1β [51,52]. When oxygen tension is normal, HIF-1α is quickly degraded [53]. Under normal oxygen concentration, HIF-1α is modified by prolyl 4 hydroxylase (PHD), which acts as a direct oxygen sensor by catalyzing the binding of molecular oxygen to a specific proline on HIF-1α [52]. The Von Hippel-Lindau cancer suppression protein binds to hydroxylated HIF-1α to activate the protein complex and targets HIF-1α for proteasome-dependent degradation following its ubiquitination [54]. Under normal oxygen conditions, asparagine residues near the C-terminus are hydroxylated by factor inhibiting HIF-1 (FIH-1), which also requires oxygen for its activity. FIH-1 reduces HIF-1α transcriptional activity by preventing the binding of p300 and cAMP response element binding protein (CREB) to HIF-α [55,56]. As PHD is inactive and HIF-1α is not hydroxylated in a low-oxygen environment, the Von Hippel-Lindau factor cannot bind and direct HIF-1α for proteasome-mediated proteolysis. Instead, HIF-1α can bind to p300 and CREB. The HIF-1α-conjugated protein is also believed to be transferred to the nucleus, heterodimerized by HIF-1β, and immediately involved in initiating transcription of target genes. With a binding site corresponding to 5′-RCGTG-3′, the HIF heterodimer transcription factor activates target genes via a hypoxia response sequence (HRE) [57]. HIF-1α binds to the HRE of VEGFA, PDGF, and transforming growth factor-alpha, inducing their expression (Figure 1) [58]. 

In addition to angiogenesis, HIF-1α activates glucose metabolism, thereby leading to acidosis in the tumor microenvironment. HIF-1α enhances the expression of glucose transporter 1, 3 and increases the uptake of glucose into cells [59]. Additionally, it cleaves glycolytic enzymes (phosphofructokinase L, hexokinase, aldolase A, and lactate dehydrogenase A) by activating ATP production and promoting glycolysis [60,61]. HIF-1α activates pyruvate dehydrogenase kinase 1, which then inactivates pyruvate dehydrogenase, resulting in suppression of the TCA cycle [62]. Thus, overexpression of HIF-1α under hypoxic conditions accelerates lactic acid production by promoting glycolysis and suppressing the TCA cycle, leading to an acidic tumor microenvironment. The latter contributes to tumor survival by conferring apoptosis resistance [63], increasing invasion and metastatic potential [64], and providing immune tolerance through T cell suppression [65].

### 4.2. Endoplasmic Reticulum Stress Signals

The endoplasmic reticulum is a protein folding and maturation site within the cell, where membrane proteins are glycosylated and secreted. Failure to mature results in the accumulation of proteins with an abnormal higher-order structure. Accumulation of such abnormal proteins causes endoplasmic reticulum stress, and the cellular responses elicited to deal with it are collectively referred to as the endoplasmic reticulum stress response. Tumor microenvironment characteristics such as hypoxia, acidosis, and glucose deprivation contribute to the activation of the endoplasmic reticulum stress pathway and promote cancer cell survival. Up-regulation of the endoplasmic reticulum molecular chaperone, BiP/GRP78 has been observed in multiple cancer cells, indicating that it is involved in their proliferation and metastasis [66]. VEGF expression is induced in the protein kinase R like endoplasmic reticulum kinase (PERK)-activating transcription factor 4 (ATF4) pathway in the endoplasmic reticulum stress environment [67]. The inositol-requiring kinase enzyme 1 alpha (IRE1α) signal also promotes cell growth in certain cancer cell types due to up-regulation of cyclin A1 via X-box binding protein 1 (XBP-1) downstream of IRE1α [68]. Expression of XBP-1 is elevated in breast cancer cells and hepatocellular carcinoma. Therefore, XBP-1 expression is believed to contribute to the survival of cancer cells by inducing BiP/GRP78 expression [69]. As in the case of PERK and IRE1α signals, ATF6 is thought to be involved in neovascularization by controlling the expression of VEGF [70]. In addition to cancer cells, tumor tissues and their microenvironments include fibroblasts, mesenchymal stem cells, and immune cells including macrophages and T cells. Cells that build these tumor microenvironments can induce angiogenesis by producing multiple growth factors, cytokines, and chemokines. Fibroblasts in tumor tissue are the major constituents of tumor stromal tissue and are said to play a vital role in cancer development. Known as cancer-associated fibroblasts (CAFs) they secrete stromal cell-derived factor 1 (SDF1). CAF-derived SDF1 not only directly stimulates cancer cell proliferation via C-X-C chemokine receptor type 4 on tumor cells, but also recruits endothelial progenitor cells towards the tumor and induces angiogenesis [71]. In colorectal cancer, angiogenesis is promoted by CAF-induced secretion of interleukin 6 and a concomitant increase in VEGF production [72]. Macrophages involved in carcinogenesis and malignancy are called tumor-associated macrophages (TAMs). Most TAMs are composed of M2 macrophages, which affect tumor development through increased immunosuppression and angiogenesis. TAMs stimulate angiogenesis directly by facilitating the production of angiogenesis promoting factors such as VEGF, and indirectly by localizing matrix metallopeptidase 9 to the tumor microenvironment. There, metallopeptidase 9 induces angiogenesis by cleaving and releasing VEGF from the matrix [73,74]. In addition, vascular endothelial cells produce Ang-2c and TAMs express its receptor, Tie2, further stimulating angiogenesis in tumor tissues [75].

## 5. Antiangiogenic Therapy

**Figure 2:**
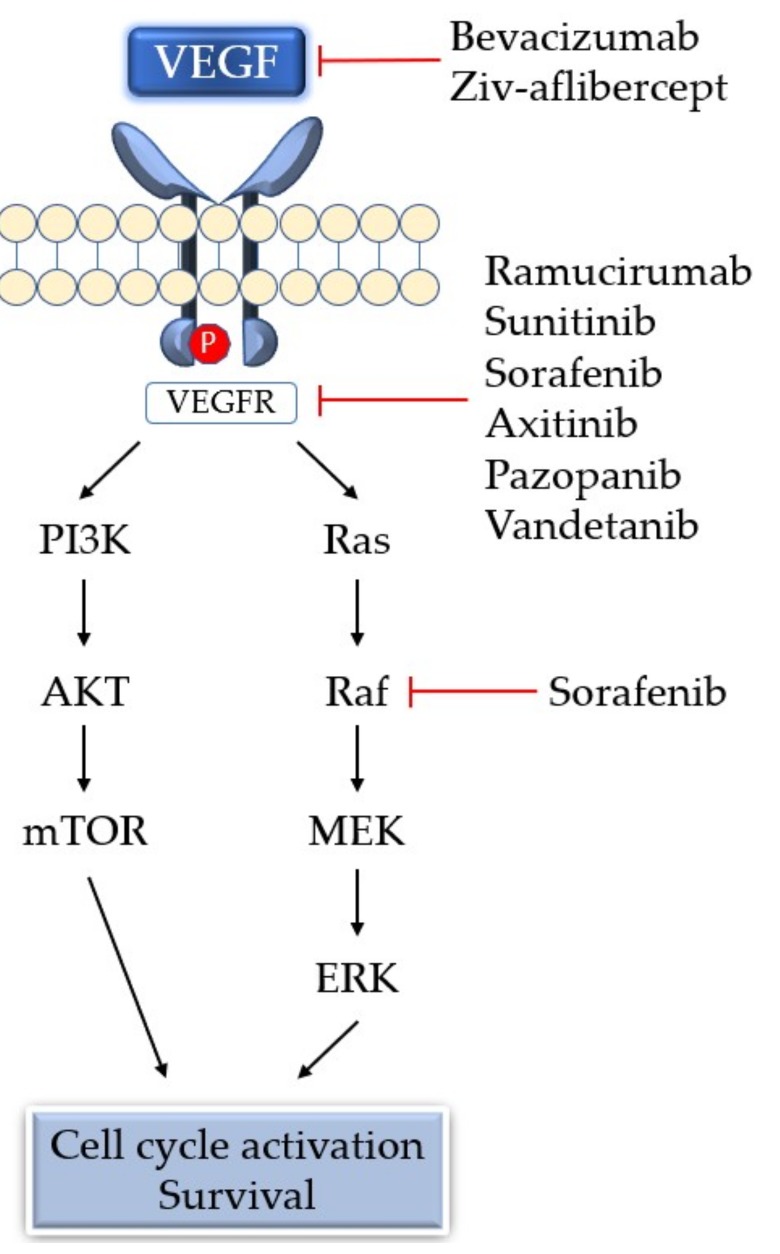
Vascular endothelial growth factor (VEGF) binds to the VEGF receptor, a receptor tyrosine kinase, leading to receptor dimerization and subsequent auto phosphorylation of the receptor complex. The phosphorylated receptor then interacts with a variety of cytoplasmic signaling molecules, leading to signal transduction and eventually angiogenesis. Examples of clinical drugs (Table 2) that inhibit the pathway are shown. PI3K, phosphoinositide-3-kinase; AKT, protein kinase B; mTOR, mechanistic target of rapamycin; MEK, MAPK/ERK kinase; ERK, extracellular signal-regulated kinase.

Clinical treatment approaches targeting tumor angiogenesis include the anti-VEGF monoclonal antibody bevacizumab, anti-VEGFR2 monoclonal antibody ramucirumab, VEGFR ligand traps (e.g., aflibercept, VEGFR, PDGFR, c-KIT), and multi-target tyrosine kinase inhibitors (e.g., sunitinib and sorafenib) [76,77] (Table 2, Figure 2). Bevacizumab, a humanized monoclonal immunoglobulin G1 antibody, is the most widely studied antiangiogenic agent that prevents VEGFA from binding to receptors, thus hindering neovascularization and the activation of signal transduction cascades [78]. After bevacizumab combined with chemotherapy was first approved by the U.S. Food and Drug Administration (FDA) in 2004, the drug was also approved by the FDA for use in non-small cell lung cancer, metastatic colorectal cancer, renal cell carcinoma (RCC), ovarian cancer, glioblastoma multiforme, cervical cancer, fallopian tube cancer, and primary peritoneal cancer [79].

Ramucirumab combined with chemotherapy has been shown to extend overall survival of gastric cancer, non-small cell lung cancer, and rectal cancer patients. In 2012, aflibercept (i.e., a peptide-antibody fusion targeting the VEGF ligand) combined with fluorouracil, irinotecan, and folinic acid was also approved by the FDA for use in colorectal cancer [80]. Sunitinib and sorafenib, which are multi-target tyrosine kinase inhibitors, have been approved as monotherapy agents based on improvement in overall survival and progression-free survival in phase III studies in metastatic-differentiated thyroid cancer, unresectable hepatocellular carcinoma, and advanced RCC [81,82,83].

## 6. Resistance Mechanism of Angiogenesis Inhibitors

Resistance to angiogenesis inhibitors develops through a variety of mechanisms such as activation of an alternate angiogenic pathway that promotes tumor angiogenesis. When VEGF and VEGFR are inhibited, other angiogenic factors such as P1GF, SDF1, Ang-1, FGF, HGF, and cytokines, are induced [84]. In preclinical models, FGF1, FGF2, Ang-1, Ephrin-A1, and Ephrin-A2 have been induced in pancreatic tumors treated with anti-VEGFR2 antibody [85]. HGF, bFGF, and P1GF levels were increased in patients with metastatic colorectal cancer before disease progression when treated with a combination of fluorouracil, irinotecan, and bevacizumab [86]. Cancers such as colorectal cancer, RCC, and neuroendocrine tumors are often highly dependent on the induction of angiogenesis by VEGF. On the opposite end, cancers that are less susceptible to anti-VEGF antibodies, such as breast cancer, pancreatic cancer, malignant melanoma or prostate cancer, use different angiogenesis mechanisms and angiogenic factors [87]. Long-term administration of angiogenesis inhibitors induces hypoxia in the tumor microenvironment by over-pruning blood vessels and up-regulates HIF-1α [88]. Angiogenesis promoting factors, such as P1GF, VEGF, Ang-1, and FGF, which are induced by HIF-1α, recruit bone marrow-derived dendritic cells (BMDCs) that mediate the growth of new blood vessels to support tumors. The presence of BMDCs in the tumor environment induces resistance to angiogenesis inhibition [89,90]. In addition to BMDCs, the hypoxic environment within the tumor promotes recruitment of regulatory T cells (Tregs), bone marrow-derived repression cells (MDSCs), and M2 TAMs.

Immune cell populations in tumors promote angiogenesis, tumor growth, epithelial-mesenchymal transition, metastasis, and immunosuppression of the tumor microenvironment [91,92]. Besides acquiring resistance to angiogenesis inhibition by inducing other cells, tumor cells have also been reported to escape the effect of angiogenesis inhibitors by adopting different neovascularization modalities, including vascular co-option and vasculogenic mimicry [93]. Vessel co-option refers to the process whereby cancer cells incorporate pre-existing vessels from surrounding tissue instead of inducing new vessel growth [94]. The main factors regulating vessel co-option are VEGF and angiopoietins. Moreover, several studies have reported an increase in vessel co-option after inhibition of angiogenesis [95]. Anti-VEGF antibody treatment in glioblastoma promotes an increase in vessel co-option, and similar phenomena have been reported in other solid tumors [96]. Vasculogenic mimicry refers to a situation, whereby tumor cells function like endothelial cells and form a blood vessel-like structure [97]. This phenomenon has been reported in malignant melanoma, sarcoma, glioma, breast cancer, and many other cancer types [98,99,100]. Preclinical studies have reported increased vasculogenic mimicry by angiogenesis inhibition therapy with bevacizumab, and the effectiveness of combining angiogenesis inhibitors with chemotherapy has been suggested.

## 7. Neovascularization and Immunity

Tumor angiogenesis and tumor immunity share a complex relationship. When exposed to hypoperfusion/vascular hyperpermeability by immature tumor neovasculature, the tumor microenvironments becomes hypoxic and VEGF is up-regulated. This induces a decrease in T cell activation by dendritic cells (DCs), a reduction in the number of intratumorally infiltrating lymphocytes, and an increase in immunosuppressive cells, all of which affect immune function [101]. Steady-state immature dendritic cells (iDCs) in vivo are superior in antigen uptake ability, but have weak T cell stimulating ability and induce immune tolerance through Treg activity. iDCs that phagocytose and process the antigen, migrate to regional lymph nodes where they convert to mature dendritic cells (mDCs) that present the antigen to T cells and activate them [102]. Although DC maturation is activated by the NF-κB pathway, the increase in VEGF due to the hypoxic environment of the tumor reduces the number of mDCs by inhibiting DC maturation through inhibition of the NF-κB pathway and suppresses immunity [103,104,105]. Furthermore, VEGF binds to VEGFR2, inhibits the T cell activation function of mDCs, up-regulates the expression of programmed cell death ligand 1 (PD-L1) (B7-H1/CD274), and suppresses the function of DCs [106]. The migration and adhesion of vascular adhesion molecules to vascular endothelial cells plays an important role in the activation of immunity by causing the accumulation of immune cells, such as macrophages, NK cells, granulocytes, B cells, and T cells [107]. VEGF promotes abnormal neovascularization and affects immune cell migration, which reduces the expression of cell adhesion molecules, such as intercellular adhesion molecule 1, vascular cell adhesion molecule 1, and E-selectin. The down-regulation of cell adhesion molecules inhibits tumor invasion by immune cells and reduces the immune response [108,109,110]. A tumor immune response is induced by tilting the quantitative and functional balance of tumor-attacking effector T cells and immunosuppressive cells to the former dominant state. The tumor hypoxic environment enhances the expression of SDF1-α and C-C motif chemokine 28, thereby inducing immunosuppressive cells such as Tregs, MDSCs, and M2 TAMs, and suppresses tumor immunity [91,92,111]. When VEGF binds to VEGFR on MDSCs, signal transducer and activator of transcription 3 signaling is activated and induces MDSC proliferation [112], VEGF also promotes an increase in Tregs in the tumor microenvironment [113,114]. Increasing the recruitment of T cells and promoting tumor invasion by angiogenesis inhibitors have shown the effect of tilting the tumor microenvironment towards immunity promotion. Bevacizumab and sorafenib induce DC maturation and improve T cell activation [115]. Inhibition of VEGF increases E-selectin expression on the tumor vascular endothelium and promotes an increase in T cell tumor invasion [116]. In the laboratory, administration of bevacizumab led to a decrease in MDSCs in the RCC mouse model, as well as a decrease in Tregs in vitro and in vivo [114,117]. A similar decrease in Tregs has been observed in RCC patients treated with sunitinib, where it correlated with overall survival [118].

## 8. Angiogenesis Inhibitors and Immunotherapy

Although the immune system is very effective in inducing an immune response against foreign antigens, malignant tumors can avoid immune surveillance via multiple mechanisms of immune tolerance. Overexpression of immune checkpoint molecules inducing immune tolerance has been demonstrated in some solid tumors, and correlates with poor prognosis [119]. Programmed-cell death-1 (PD-1 is a checkpoint molecule expressed on the outer surface of NK cells, B cells, DCs, monocytes, and CD4 + and CD8 + T cells [120]. When PD-1 is expressed by T cell stimulation and binds to PD-L1 and PD-L2 on antigen-presenting cells and some cancer cells, the Ras/MAPK/ERK kinase/ERK pathway and PI3K/AKT pathway are inhibited and inactivate T cells [120]. PD-L1 is expressed in cancers of tissues such as the lung, colon, ovaries, as well as in malignant melanoma and its expression is enhanced by the inflammatory cytokine interferon gamma [121]. Additionally, activation of HIF-1 in a hypoxic environment within a tumor leads to elevated expression of PD-L1 in cancer tissue [122]. In other words, cancer cells escape immune surveillance by inactivating locally accumulated T cells through the PD-1/PD-L1 pathway. Immunological checkpoint inhibitors such as nivolumab and pembrolizumab, which are PD-1 inhibitors, and atezolizumab, which is a PD-L1 inhibitor, promote the antitumor activity of T cells by blocking these pathways, and are clinically effective in several cancer types [122]. As mentioned in the previous section, angiogenesis inhibitors and immunological checkpoint inhibitors are expected to have a combined immunostimulatory effect. Increased infiltration of CD4 + and CD8 + T cells, in addition to macrophages, into the tumor space and increased expression of PD-L1 in the tumor by co-administration of bevacizumab and sunitinib have been shown in the RCC mouse model [123]. Additionally, a decrease in MDSCs in tumor tissue and an increase in PD-1 expression in tumor infiltrating lymphocytes have been observed in RCC patients treated with sunitinib [124]. Combination therapy with atezolizumab and bevacizumab resulted in an increase in CD8 + T cells and major histocompatibility complex 1 in the tumor, as well as up-regulation of chemokines and down-regulation of genes associated with neovascularization in patients with metastatic RCC [125]. Several phase III studies on the combined treatment of angiogenesis inhibitors and immunity checkpoint inhibitors are in progress, and the results of these preclinical and clinical trials are listed in Table 3. Two phase III trials have shown that a combination therapy of atezolizumab and bevacizumab is effective and tolerable. Comparisons of combination chemotherapies (carboplatin and paclitaxel) and atezolizumab + bevacizumab in untreated non-small cell lung cancer have shown better survival (response rate, progression-free survival, and overall survival) in the atezolizumab + bevacizumab group than in the chemotherapy + bevacizumab group. Subgroup analysis of the low PD-L1 expression group, the group with low effector T cell gene expression, and the liver metastases group also shows similar results [126]. In a study of patients with metastatic RCC characterized by ≥1% PD-L1 expression, the combination therapy (bevacizumab and atezolizumab) group had a longer progression-free survival than the sunitinib monotherapy group [127].

## 9. Conclusions

In this review, we have discussed the mechanism of tumor angiogenesis as well as antiangiogenic therapy from the perspective of the tumor microenvironment. Although angiogenesis inhibitors have been used in combination with chemotherapy for more than 10 years, resulting overall survival has increased by only a few months and resistance to treatment has often developed rapidly. Angiogenesis inhibitors have failed to improve overall survival in some cancers such as breast cancer. These findings highlight the complexity of the pathways involved in tumor neovascularization and raise questions about the effective use of antiangiogenic therapy in cancer treatment. Therefore, we need to better understand the role of neovascularization in different cancers and how they avoid the effects of antiangiogenic therapy. A combination therapy with angiogenesis inhibitors and immunotherapy effectively enhances the benefits of angiogenesis inhibitors and represents the most promising path ahead.

## Figures and Tables

**Table 1:** Endogenous regulators of angiogenesis.

Activators	Functions	Inhibitors	Functions
**Vascular endothelial growth factor family**	Induction of angiogenesis, enhancement of vascular permeability	**Angiopoietin-2**	Antagonist of Ang1
**Epidermal growth factor**	Promotes growth of vascular endothelial cells	**Thrombospondin-1,2**	Inhibits endothelial migration, growth, adhesion and survival
**Fibroblast growth factor**	Induction of angiogenesis	**collagen**	Substrate for MMPs
**Platelet-derived growth factor**	Involved in migration of vascular endothelial cells	**Endostatin**	Inhibits endothelial survival and migration
**Angiopoietin-1**	Stabilization of vascular endothelium	**Angiostatin**	Suppresses tumor angiogenesis
**Transforming growth factor**	Production of extracellular matrix	**TIMPs**	Suppresses pathological angiogenesis
**Ephrin**	Control of blood vessel and lymph duct formation	**Platelet Factor-4**	Inhibits binding of bFGF and VEGF
**Matrix metalloproteinase**	Degradation of extracellular matrix, activation of angiogenesis inducing factor	**Vasostatin**	Inhibits endothelial growth

**Table 2:** Angiogenesis inhibitors approved by FDA.

Drug	Target Molecule	Approved Disease
**Bevacizumab**	Anti-VEGF monoclonal antibody	mCRC, NSCLC, mRCC, ovarian cancer, malignant glioma, advanced cervical cancer, fallopian tube cancer, primary peritoneal cancer
**Ramucirumab**	Anti-VEGFR2 monoclonal antibody	Advanced gastric or gastroesophageal junction adenocarcinoma, NSCLC, advanced colorectal cancer
**Ziv-aflibercept**	Soluble decoy of VEGFR	Metastatic colorectal cancer
**Sunitinib**	TKI: VEGFR, PDGFR, FLT3, KIT	RCC, Gastrointestinal stromal tumor, pancreatic neuroendocrine tumor
**Sorafenib**	TKI: VEGFR, PDGFR, FLT3, KIT, Raf	RCC, unresectable hepatocellular carcinoma, metastatic or recurrent thyroid carcinoma
**Axitinib**	TKI: VEGFR, PDGFR, KIT	Advanced RCC
**Pazopanib**	Multiple targeted receptor TKI	RCC, Advanced soft tissue sarcoma
**Vandetanib**	TKI: VEGFR, EGFR, RET	Unresectable or metastatic medullary thyroid cancer

**Table 3:** Selected ongoing phase III clinical trials involving anti-angiogenic inhibitors combined with cancer immunotherapy.

Tumor Type	Combination Drugs	Study Status	NCT ID
**Stage IV NSCLC**	Atezolizumab+Carboplatin+paclitaxel+Bevacizumab	Active, not recruiting	NCT02366143
**Advanced RCC**	Bevacizumab+Atezolizumab	Active, not recruiting	NCT02420821
**Advanced RCC**	Avelumab+Axitinib	Active, not recruiting	NCT02684006
**Advanced RCC**	Lenvatinib/Everolimus or Lenvatinib/Pembrolizumab	Recruiting	NCT02811861
**Recurrent OC, FTC, or PPC**	Pegylated Liposomal Doxorubicin+Atezolizumab+Bevacizumab	Recruiting	NCT02839707
**RCC**	Pembrolizumab+Axitinib	Active, not recruiting	NCT02853331
**Late relapse OC**	Atezolizumab+Chemotherapy+Bevacizumab	Recruiting	NCT02891824
**OC,FTC,or PPC**	Atezolizumab+Carboplatin+paclitaxel+Bevacizumab	Recruiting	NCT03038100
**Early relapse OC**	Atezolizumab+Bevacizumab+Chemotherapy	Recruiting	NCT03353831
**Locally Advanced or Metasatatic HCC**	Atezolizumab+Bevacizumab	Recruiting	NCT03434379
